# Relationship between clinical and laboratory features with infecting dengue virus serotypes in a sample of dengue suspected adult patients from 2015-2017 in Sri Lanka

**DOI:** 10.1016/j.jcvp.2022.100112

**Published:** 2022-11

**Authors:** Chandana Wijesinghe, Afzal A Jabeer, Bushran N Iqbal, Faseeha Noordeen

**Affiliations:** a Postgraduate Institute of Science, University of Peradeniya, Peradeniya 20400, Sri Lanka; b Diagnostic and Research Virology Laboratory, Department of Microbiology, Faculty of Medicine, University of Peradeniya, Peradeniya 20400, Sri Lanka

**Keywords:** Dengue, Clinical and laboratory features, Dengue virus serotypes, Adult dengue patients, Sri Lanka, DENV, Dengue virus/es, BHT, Bed head ticket, ORF, Open reading frame, MOH, Medical officer of health, WBC, White blood cells, WHO, World Health Organization

## Abstract

Dengue is a major viral disease affecting the tropics. Although previous research has focused on the relationship between the infecting dengue virus (DENV) serotypes and disease severity, less work has been done on the relationship between the clinical and laboratory features and the infecting DENV serotypes in Sri Lanka. We evaluated the relationship between the clinical and laboratory features and the infecting DENV serotypes in adult patients with clinically suspected dengue admitted to the Base Hospital, Mawanella, Sri Lanka from December 2015 to March 2017.

Blood samples of 200 dengue suspected patients were tested for the infecting DENV serotypes using a reverse transcription polymerase chain reaction with primers targeting the envelope region of the virus. Relationship between the infecting DENV serotypes with clinical and laboratory features was assessed using Z score and paired *t* tests.

Of the 200 patients tested, 39 (19.5%) were positive for DENV, any of the four DENV serotypes alone or in combination. The highest number of infections was noted with DENV-2 (n=18, 46.1%). Fever (*P*=0.000) and rash (*P*=0.017) were frequently noted in DENV negative patients while bleeding (*P*=0.012) was more frequently noted in DENV serotype positive patients. Platelet count of <100,000 μl^−1^ was significantly associated with DENV serotype positivity (*P*=0.000). Platelet count of <100,000 μl^−1^ (*P*=0.035) and haemoglobin (Hb) of >13mgdl^−1^ (*P*=0.016) were noted in 15 of the 18 DENV-2 positive patients.

Clinical and laboratory features of severe dengue with bleeding manifestations, low platelet counts and high Hb were noted in DENV-2 infections.

## 1 Introduction

Dengue is one of the mosquito-borne viral diseases in tropical and subtropical regions and poses a high burden in many regions worldwide including Sri Lanka. Although very few countries reported dengue epidemics before 1970, the disease is now endemic in regions of Africa, America, Western Pacific, Eastern Mediterranean and South-East Asia and annually, 390 million dengue infections are being reported from more than 100 countries [1].

Dengue has a broad spectrum of clinical presentations, most patients recover following a self-limiting non-severe clinical course, a small proportion progress to severe dengue. Early recognition of dengue is a challenge as initial symptoms are non-specific, viraemia may be short lived, and serological tests confirm dengue late in the course of illness [2]. Prompt diagnosis during the febrile phase helps appropriate patient management.

Dengue viruses (DENV) have been circulating in Sri Lanka since the 1960s, and the first dengue outbreaks were reported in 1965, accounting for 26 cases and 6 deaths. The initial large severe dengue outbreak in Colombo, the capital of Sri Lanka, occurred in 1989, with 206 clinically diagnosed cases and 20 deaths, followed by in 1990, 1,080 cases with 60 deaths. In 2017, the island reported the largest ever outbreak with a total number of 186,101 cases and 440 deaths, which coincided with the re-emergence of DENV-2. Although fewer cases were reported in 2018 (51,659 cases) compared to 2017, there was again another surge in 2019, with 105,049 cases reported [3], [4], [5].

Laboratory testing plays a vital role in the confirmation of dengue as the infection produces a wide range of symptoms, many of which are non-specific. Laboratory diagnosis of dengue involve direct detection of viral nucleic acid by reverse transcription PCR (RT-PCR) or dengue NS1 antigen by an immuno-assay or serological tests for detection of anti-DENV IgM/IgG antibodies [6], [7], [8]. In addition to specific diagnostic tests to demonstrate DENV infection, full blood count including white blood cell count, platelet count and haematocrit are of importance to align with clinical features to diagnose and determine the severity of the disease [9].

Sri Lanka is a dengue endemic country with the occurrence of periodic epidemic outbreaks. All four DENV serotypes have been co-circulating in Sri Lanka for more than three decades. DENV-3 contributed to the epidemics in the latter part of the1989 to 2008 outbreak, while DENV-1 was responsible for the 2009 dengue epidemic [4,10]. From 2012 to 2014, DENV-1 was the predominant serotype contributing to the outbreaks with lesser involvement of DENV-4 [3]. In 2017, the predominant circulating serotype was DENV-2, and this serotype contributed to the largest ever dengue outbreak recorded in Sri Lanka [11].

Previous studies have analyzed the clinical and epidemiological features of dengue including the factors responsible for severe dengue [12], [13], [14], [15], however, only a few studies have been carried out to compare the relationship between clinical and laboratory features with the infecting DENV serotypes [16], [17], [18], [19], [20]. Hence, we analyzed the relationship between clinical and laboratory features with the infecting DENV serotypes in a sample of clinically suspected adult dengue patients in a tertiary care hospital in a high dengue risk area of Sri Lanka.

## 2 Materials and Methods

### 2.1 Study design

Ethical clearance for the study was obtained from the Ethics Review Committee of the Medical Research Institute, Ministry of Health, Colombo, Sri Lanka and the Ethical Review Committee of the Postgraduate Institute of Science, University of Peradeniya, Sri Lanka. This is a prospective study carried out for 16 months from December 2015 to March 2017, in the Mawanella medical officer of health (MOH) area. Blood samples of dengue suspected patients, data on clinical and laboratory features were collected from the Base Hospital, Mawanella. A conventional two step reverse transcription polymerase chain reaction (RT-PCR) was performed using the Swift MaxPro Thermal Cycler (Esco Healthcare, Singapore) using primers targeting the capsid region of the virus to confirm DENV infection in dengue suspected patients [7]. DENV-positive samples were then subjected to DENV serotyping using primers targeting the envelope region of the virus [7]. The relationship between infecting DENV serotypes and clinical and laboratory features were then evaluated using Z score and paired *t* tests.

### 2.2 Data collection and selection of patients

Adult patients admitted to the Base Hospital, Mawanella with suspected dengue from December 2015 to March 2017 were recruited for the study. Considering the cost and feasibility, 200 adult patients were selected by including every second patient with suspected dengue during the study duration. Patients who were severely ill and could not consent, those who did not consent, and children were excluded from the study. With the written consent of the selected patients, an additional 3-5 ml of blood was collected when blood had been collected for routine investigations. The serum was separated and sent to the Virology Laboratory, Department of Microbiology, Faculty of Medicine, University of Peradeniya for RT-PCR testing to identify DENV. Then the next RT-PCR was done on DENV positive samples to identify the infecting DENV serotype.

### 2.3 Clinical and non-specific laboratory findings

Bed head tickets (BHTs) of recruited patients were collected from the Hospital and studied by the research candidate and a data collection team. Some common clinical features were selected based on the literature for evaluation. During the scrutinization of BHTs of the patients, presence of selected clinical features was noted and the research candidate recorded the laboratory findings and entered to the data sheet.

### 2.4 Statistical analysis

Z score and paired *t*-tests were used to identify the relationship between the infecting DENV serotypes and clinical and laboratory features. A relationship was considered significant when the *P* value was <0.05.

## 3 Results

### 3.1 Relationship between clinical features and infecting DENV serotypes

Of the 200 clinically suspected adult dengue patients, 39 (19.5%) were positive for DENV and these 39 patients were positive for any of the four DENV serotypes alone or in combination (Table 1). DENV-2 and DENV-3 were detected in more patients than other serotypes, and DENV-4 was the least common infecting serotype. DENV-2 was detected in 18 of the 39 patients as mono-infection or co-infections, constituting 46.1%. Of the 7 patients with bleeding, 3 were mono-infections with DENV-2; 2 were mono-infections with DENV-3; 2 were co-infections with DENV-2 & DENV-1 and DENV-2 & DENV-4. Overall, 5 out of 7 patients with bleeding tendencies were infected with DENV-2 alone or in combination.

**Table 1:** Distribution of DENV serotypes from December 2015 to March 2017 in adult dengue patients at Base Hospital, Mawanella, Sri Lanka (n=39).

	**DENV serotype/ serotypes combination**						
	**DENV-1**	**DENV-2**	**DENV-3**	**DENV-4**	**DENV-1 + DENV-2**	**DENV-2 + DENV-3**	**DENV-2 + DENV-4**
Number of DENV positive patients =39	6	14	12	3	1	1	2
Number of patients with bleeding tendencies	0	3	2	0	1	0	1

Of the 200 dengue suspected patients recruited for the study, 75% were males. Of the 39 DENV-positive patients, 59.5% were males. Fever was noted on admission in 76.9%, and 97.5% of DENV positive and DENV negative patients, respectively. The difference between fever & DENV negativity and fever & DENV positivity was statistically significant (*P*=0.000). The mean duration of fever on admission to the hospital between DENV-positive and DENV-negative patients were 4.4 and 3.8 days, respectively. The difference in the mean duration of fever between DENV-positive and DENV-negative patients was not statistically significant (*P*>0.05).

Fever, headache and rash were noted as the common clinical features among DENV negative patients (Table 2). A statistically significant difference was noted between DENV positivity and bleeding manifestations (*P*=0.012), and it was high in DENV-2 infected patients (5/7; 71.4%) (Table 1). Headache was a complaint of 33/39 (84.6%) of DENV positive and 148/164 (90.2%) of DENV negative patients. Fever was reported by 30/39 (76.9%) and 160/164 (97.5%) of DENV positive and negative patients, respectively. Rash was noted in 1/39 (2.5%) and 6/164 (3.6%) of DENV positive and negative patients, respectively. Bleeding was noted in 7/39 (17.9%) and 10/164 (6.1%) of DENV positive and negative patients, respectively (Table 2).

**Table 2:** Distribution of clinical features among DENV positive and negative patients at Base Hospital, Mawanella, Sri Lanka (n=200).

**Clinical features**	**Number (%) in DENV positive patients n=39**	**Number (%) in DENV negative patients n=164**	***P* value**
Fever	30 (76.9)	160 (97.5)	*P=0.000
Headache	33 (84.6)	148 (90.2)	*P*=0.673
Rash	1 (2.5)	6 (3.6)	*P=0.017
Bleeding	7 (17.9)	10 (6.1)	*P=0.012

⁎ Statistical significance at *P* value <0.05.

### 3.2 Relationship between laboratory findings and infecting DENV serotypes

As shown in Table 3, the cut-off points for selected laboratory parameters were taken from the WHO as well as Sri Lankan dengue guidelines [21,22]. These parameters included white blood cell count (WBC) below 4,000 mm^−3^, platelet count below 100,000 μl^−1^, packed cell volume (PCV) above 50 mlh^−1^ and Hb level above 13 mgdl^−1^ as markers of disease severity.

**Table 3:** Laboratory features of DENV positive and negative patients at Base Hospital, Mawanella, Sri Lanka (n=200).

**Laboratory parameters**	**Number (%) in DENV positive patients (n=39)**	**Number (%) in DENV negative patients (n=161)**	***P* value**
WBC <4,000 mm^−3^	31 (79.48%)	118 (73.29%)	*P*=0.076
Platelet count <100,000 μl^−1^	25 (64.1%)	27 (16.77%)	*P=0.000
Packed cell volume PCV>50 mlh^−1^	0 (00%)	0 (00%)	0
Hb>13 mgdl^−1^	17 (43.58%)	101 (62.73%)	*P*=0.073

⁎ Statistical significance at *P* value <0.01.

Based on the guidelines, there was no significant difference between the percentage of DENV-positive and negative patients when considering WBC count of <4,000 mm^−3^. However, the percentage of patients with platelet count of <100,000 μl^−1^ in DENV-positive patients was significantly higher than that in the DENV-negative patients. There was no statistically significant difference in the percentage of patients with Hb of >13 mgdl^−1^ between DENV-positive and DENV-negative groups (Table 3).

Of the 39 DENV positive patients, 18 were infected with DENV-2 as mono-infections or co-infections (Table 4), and 14 out of 18 of DENV-2 (77.77%) infected patients had a platelet count of <100,000 µl^−l^. However, 11 out of 21 patients (52%) with other DENV infections had platelet counts of <100,000 µl^−1^. The difference between the number of DENV-2 infected patients with platelet counts of <100,000 µl^−1^ and the number of other DENV infected patients with platelet counts of <100,000 µl^−1^ was statistically significant (*P*=0.035). Fifteen out of 18 DENV-2 (83%) and 7 out of 21 other DENV serotype positive (33%) patients had Hb of >13 mgdl^−1^ and this difference was also statistically significant (*P*=0.016). More DENV-2 positive patients had bleeding manifestations than those positive for other DENV serotypes (Table 4).

**Table 4:** Distribution of clinical/laboratory features of severe dengue among DENV-2 and other DENV infected patients at Base Hospital, Mawanella, Sri Lanka (n=39).

**Clinical/ laboratory features**	**Proportion of severe dengue feature in DENV-2 and other DENV infected patients**		
	**DENV-2**	**Other DENV types**	***P* value**
Bleeding manifestation	5/18	2/21	*P*=0.045
Low (<100,000µl^−1^) platelet count	14/18	11/21	*P=0.035
Hb>13mgdl^−1^	15/18	7/21	*P=0.016

⁎ Statistical significance at *P* value <0.05.

## 4 Discussion

Research carried out in dengue endemic Asian countries including Sri Lanka and dengue endemic non-Asian countries like Brazil, has analysed the relationship between clinical and laboratory findings and infecting DENV serotypes. Some studies noted a clear relationship between clinical and laboratory findings and infecting DENV serotypes whereas others did not. The present study is one of the few studies done in Sri Lanka to assess the relationship between infecting DENV serotypes with clinical and laboratory findings.

In the current study, almost 20% of the patients were positive for DENV serotypes and all four DENV serotypes were detected among DENV-positive patients during the 16 months’ study period. This study also identified four co-infections between different DENV serotypes. Based on a previous study [23], when co-circulation of DENV serotypes expands in an endemic setting, DENV co-infections also increase. The proportion of infection with DENV-2 (n=14) followed by DENV-3 (n=13), DENV-1 (n=6) and DENV-4 (n=3) in our study sample reflects the epidemiology of dengue in Sri Lanka. In 2017 in the study area, DENV-2 was the main circulating serotype which resulted in the largest ever outbreak in the island [11]. Similar findings on circulating DENV serotypes have been reported in Malaysia [16].

Taken the findings of the current study, the mean duration of fever on admission to the hospital between DENV-positive and DENV-negative patients did not differ significantly (4.4 vs 3.8 days). Fever was observed on admission in 97.5% DENV negative patients and 76.9% of DENV positive patients. Body temperature subsides around days 4 and 5 from the onset of symptoms in dengue [21] and most of the dengue suspected patients in this study were admitted to the Hospital on fever day 4 or 5 and this may be the reason for more patients not complaining of fever on admission. DENV NS1 or DENV RNA detection is suitable for the laboratory diagnosis of dengue before fever day 4 and anti-DENV IgM is suitable for diagnosis after fever day 5 and there is a window period from day 4 to 6, in which both parameters may become undetectable [24]. The WHO recommendation for virus detection is up to fever days 4-5 [21]. In the present study, as explained earlier also, the mean duration of fever on admission and delays in blood collection might have contributed to the detection of DENV in a limited number of patients. This finding is important clinically as sometimes dengue patients may be ignored when there is no fever on admission. Bleeding tendencies were significantly higher among DENV positive patients. Notably, 27.7% of DENV-2 positive patients had bleeding tendencies. These findings are in agreement with studies done in different geographical regions [25], [26], [27], where DENV-2 infections have been associated with bleeding tendencies and severe dengue.

Of the laboratory findings assessed, platelet count of <100,000 µl^−1^, a risk factor for bleeding and a feature of severe dengue was significantly higher among DENV positive patients than DENV negative patients. These findings are compatible with the findings of an Australian study in which travellers returning from dengue-endemic countries with dengue had low platelet and WBC counts when compared to DENV negative fever patients [28]. Additionally, in the current study a significant percentage of DENV-2 positive patients had platelet counts of <100,000 µl^−1^ compared to those infected with other DENV serotypes. Although there was no significant difference in the number of patients with Hb of >13 mgdl^−1^ between DENV positive and negative groups, it was significantly higher among DENV-2 infected patients than those infected with other DENV serotypes. Similar findings with DENV-2 infection have been reported elsewhere by other studies [25], [26], [27]. Ekanayake *et al* [22] report that DENV positive patients have a lower WBC and platelet counts than others. In the present study, there was no difference in WBC count between DENV positive and negative groups. DENV positive group had a higher Hb% level than the DENV negative group. As previous studies [24,29] suggest co-infection may have a protective effect on the pathogenesis of DENV infection and disease severity. The same was true for the co-infected patients in the current study, however, co-infection sample size was small to make a strong conclusion on the protective effect of co-infections from these findings.

The predominant DENV serotype circulating in Sri Lanka changed to DENV-2 in the 2017 outbreak, and more infected patients had features of severe dengue [30]*.* Taken the clinical and laboratory findings of DENV-1 infected patients of 2015-2016 and DENV-2 infected patients of 2016-2017, lower platelet count, high risk of plasma leakage and severe dengue have been noted in DENV-2 infected patients than DENV-1 infected patients.

In the present study, patients were selected from a single time frame. However, the current findings show the tendency of the DENV-2 to cause severe dengue. In contrast, Senaratne *et al* [7] from Kandy, Sri Lanka and Wardhani *et al* [31] from Surabaya, Indonesia did not note any relationship between infecting serotype and clinical or laboratory findings. DENV-2 associated features of severe dengue including plasma leakage and low platelet count have been noted by some other studies [32,33]. Young *et al* [27] and Rocha *et al* [34] reported a correlation between DENV-1 infections and severe dengue.

The present study reiterates the relationship between DENV-2 infection and the risk of developing severe dengue. These findings support the postulate that virus serotype is associated with disease severity. Furthermore, the results of the present study emphasize the importance of identifying the circulating DENV serotypes in an area for better patient management by predicting clinical and laboratory features for possible complications.

## 5 Conclusion

Mean days of illness of 4-5 in most patients on admission may have contributed to relatively low percentage of (19.5%) DENV positivity in the study sample of dengue suspected patients. Some patients did not have fever on admission and this may lead to misdiagnosis of dengue. DENV-2 was the most predominant serotype detected from December 2015 to March 2017 in the study area. Clinical and laboratory features of severe dengue like bleeding manifestations, low platelet counts (<100,000µl^−1^) and high Hb (>13mgdl^−1^) were associated with DENV-2 infections.

## Funding

The work was supported by the World Health Organization / TDR. The funding body did not have a role in the design of the study, collection of samples, analysis, interpretation of data and writing the manuscript.

## Authors’ contribution

Chandana Wijesinghe: Data curation, Funding acquisition, Writing – original draft.

Afzal A Jabeer: Data curation, Writing – review & editing.

Bushran N Iqbal: Data curation, Formal analysis, Writing – review & editing.

Faseeha Noordeen: Conceptualization, Supervision, Project administration, Visualization, Writing – review & editing.

## Declaration of Competing Interest

The authors declare no conflict of interest.
